# Start, speed, and direction of secondary forest succession

**DOI:** 10.1126/sciadv.aed5470

**Published:** 2026-08-07

**Authors:** Tomonari Matsuo, Iris Hordijk, Lucy Amissah, Susan G. W. Laurance, Miguel Martínez-Ramos, Jorge A. Meave, Frans Bongers, Masha T. van der Sande, Jazz Kok, Lourens Poorter

**Affiliations:** ^1^ Forest Ecology and Forest Management Group, Wageningen University & Research, P.O. Box 47, 6700 AA Wageningen, Netherlands.; ^2^ Council for Scientific and Industrial Research–Forestry Research Institute of Ghana, Kumasi, Ghana.; ^3^ CSIR College of Science and Technology, P.O. Box M 32, Accra, Ghana.; ^4^ Centre for Tropical Environmental and Sustainability Science (TESS), James Cook University, Cairns, QLD, Australia.; ^5^ Instituto de Investigaciones en Ecosistemas y Sustentabilidad, Universidad Nacional Autónoma de México, CP 58190 Morelia, Michoacán, México.; ^6^ Departamento de Ecología y Recursos Naturales, Facultad de Ciencias, Universidad Nacional Autónoma de México, Coyoacán, 04510 Mexico City, Mexico.

## Abstract

Succession is a foundational concept in ecology, describing changes in populations, communities, and ecosystems over time following a disturbance. Predicting successional pathways is challenging because of high spatiotemporal variability. Here, we present a unifying mechanistic framework that integrates three core components of succession (start, speed, and direction) that define successional pathways. We monitored the first 5 years of forest succession in recently abandoned agricultural fields in six tropical landscapes across three countries that differ in land-use intensity and between dry and wet forests. We found that (i) secondary succession typically started with woody legacies and progressed slowly but directionally toward structurally complex, biodiverse forests, and (ii) the start and speed were greater in areas with short-duration, low-intensity (nonmechanized) land use associated with more biological legacies. This framework advances mechanistic understanding of succession by disentangling the start, speed, and direction, and provides a tool for designing effective forest restoration strategies.

## Teaser

Decomposing succession into start, speed, and direction reveals how secondary forests recover after disturbance.

## INTRODUCTION

Succession is a fundamental concept in ecology, describing changes in populations, communities, and ecosystems over time on new substrates (primary succession) or following disturbance (secondary succession) (*1*–*4*). In the Anthropocene, secondary succession has become increasingly important because global climate and land-use changes intensify disturbance regimes, driving widespread vegetation changes, with large consequences for biodiversity conservation, ecosystem functioning, and human well-being (*5*–*8*). Understanding succession is therefore urgent for predicting and mitigating the impacts of global changes on nature and people (*9*). Yet, predicting successional pathways remains challenging because of substantial variation across and within sites (*1*, *10*, *11*), much of which emerges during the first years of succession (*10*, *12*–*14*). To explain this variation, successional frameworks have been developed that primarily focus on how socioecological drivers such as macroclimate, landscape context, land-use history (*4*, *9*, *12*), and feedback loops between plants and environment (*15*) shape successional dynamics. Here, we propose a unifying mechanistic framework that allows us to quantitatively compare successional pathways by integrating three complementary key components of succession (start, direction, and speed), which together define long-term trajectories. Our framework advances the understanding of succession, enables quantitative comparisons of successional pathways across systems, and supports the development of effective forest restoration strategies.

The start, direction, and speed of succession capture three complementary aspects of forest succession. The start refers to the initial vegetation at the time of land abandonment. This stage has often been overlooked because early ecological theories assumed that succession was mainly driven by natural processes, with little attention to the human context (*3*, *16*, *17*). Yet, secondary succession on abandoned agricultural land is the outcome of a socioecological process (*18*), where the type, duration, and intensity of previous land use strongly determines the availability of propagules, seedlings, and remnant trees that initiate succession (*9*, *19*, *20*).

The direction refers to the orientation of change in vegetation structure, diversity, and composition over time. This component captures whether successional change proceeds in a similar direction across plots (convergence or parallel pathways) or in multiple directions (divergence) within the landscape. Classical theories viewed succession either as a deterministic process, converging toward a stable climax state (*3*) or as a stochastic process with divergent trajectories shaped by species-specific responses to environmental conditions (*16*). It is now widely recognized that both deterministic and stochastic processes operate simultaneously (*10*, *15*), yet few studies have explicitly quantified the orientation of successional trajectories across and within sites using standardized long-term monitoring data (*10*).

The speed refers to the rate of change in vegetation attributes, which is primarily shaped by climatic conditions and human land use (*19*, *21*). Climatic conditions set the upper boundaries to vegetation productivity because wetter climates generally provide longer growing seasons and more favorable environmental conditions for plant growth, recruitment, and rapid recovery (*11*, *22*, *23*). Furthermore, land-use type and intensity leave lasting biological legacies, such as resprouting individuals and seed(ling) banks that initiate succession (*19*–*21*) and remnant trees that serve as regeneration nuclei (*24*). Therefore, these legacies not only determine the starting conditions but also the subsequent rate of forest recovery.

Despite the important roles of these three components in shaping succession, they have rarely been assessed simultaneously partly because of a lack of a conceptual and analytical framework that quantifies start, speed, and direction within a unified multivariate space, and partly because few successional studies have established plots right after land abandonment or monitored early vegetation dynamics over time.

**Fig. 1.**
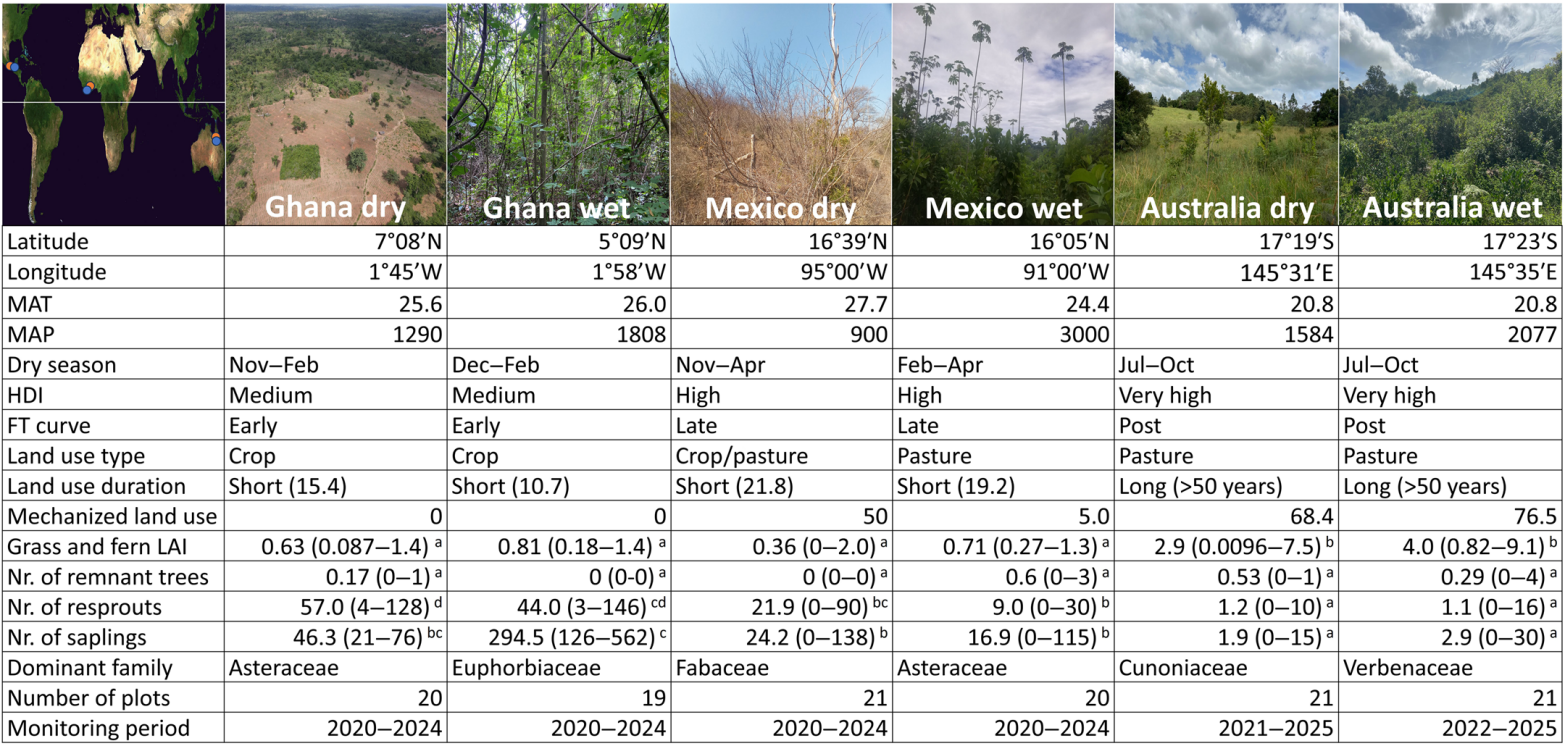
Overview of the six study sites with site characteristics. The map shows the location of the six study sites in wet evergreen forests (blue circles) and dry semideciduous forests (orange circles) across Ghana, Mexico, and Australia. The equator is indicated by a white line. At the top, representative site photographs are shown. The accompanying table summarizes key site characteristics: latitude and longitude; mean annual temperature (MAT, °C); mean annual precipitation (MAP, millimeter per year); dry season duration (mean monthly precipitation <100 mm) (*21*, *30*, *35*); human development index (HDI: composite index of life expectancy, education, and income per capita) (*72*); position along the forest transition curve (FT curve: stage of forest cover change along the forest transition trajectory, from net deforestation to forest recovery) (*73*); primary land-use (LU) type (pasture versus crop); land-use duration (average years of previous land use across plots estimated from interviews with landowners and complemented by historical aerial photography); land-use intensity (percentage of plots where mechanized agriculture was used for field preparation); leaf area index (LAI, m^2^ m^−2^) of grasses and ferns between 0- and 2-m height along four 25-m transects at land abandonment (stand age = 0); number of resprouting individuals (DBH ≥ 1 cm) per plot (625 m^2^) at land abandonment; number of saplings (DBH <1 cm, height ≥30 cm) per plot along four 1 m–by–25 m transects (100 m^2^) at land abandonment; and the most abundant woody plant family based on the permanent plot data (dominant family), number of plots, and monitoring period. Different letters in the table indicate significant differences among landscapes based on generalized linear models with a negative binomial distribution (saplings), and linear models with square-root–transformed response variables (resprouting individuals and LAI of grasses and ferns). Photo Credit: Iris Hordijk, Wageningen University & Research.

We apply this framework to 122 permanent young secondary tropical forest plots established in recently abandoned agricultural fields across six human-modified landscapes. The landscapes are in three tropical countries (Australia, Ghana, and Mexico) located in three continents (Australia, Africa, and North America). The countries differ in biogeographic conditions, with distinct floristic compositions, and in socioeconomic development (the highest in Australia, intermediate in Mexico, and lowest in Ghana), resulting in contrasting agricultural land-use practices (Fig. 1). For example, nonmechanized crop cultivation is more common in Ghana, whereas prolonged mechanized pasture is more common in Australia (Fig. 1). These differences in land use strongly influence the type and quantity of biological legacies at land abandonment (*19*, *20*), with greater woody legacies (e.g., resprouting individuals and saplings) in Ghana and Mexico, and greater herbaceous legacies (e.g., more abundant grasses and ferns) in Australia (Fig. 1). Within each country, we included two contrasting forest types (dry and wet forests) that represent the main forest types in lowland tropical forests and differ strongly in environmental conditions (e.g., annual precipitation and length of the dry season) (*25*).

We address two overarching questions: (i) How do the start, speed, and direction of succession vary across countries differing in land-use history and between dry and wet tropical forests? (ii) How do the three successional components interrelate and result in distinct, dominant successional pathways? We propose the following four hypotheses:

1. The start of succession will increase with decreasing land-use intensity as shorter-duration and nonmechanized land use retain greater quantities of woody biological legacies at land abandonment (Fig. 1) and, thus, is expected to be highest in Ghana followed by Mexico and Australia.

2. The speed of succession will decrease with increasing land-use intensity as prolonged and mechanized land use promotes a high abundance of competitive grass and ferns that hinder tree establishment and slow recovery (*26*, *27*) and therefore will follow the same cross-country pattern to that of start. The speed of succession will be faster in wet forests than in dry forests because of a longer growing season and more favorable environmental conditions (*22*, *23*).

3. The direction of succession will be more consistent when land-use intensity is either low (because of more intact forest landscapes and woody legacies, such as in Ghana) or high (because of little woody legacies and strong resource competition with herbaceous vegetation, such as in Australia). The direction will be more variable at intermediate land use intensity because of greater heterogeneity in land use, such as in Mexico (*13*). The direction will be more variable in wet than in dry forest because the greater species pool and more complex structure result in a greater variation in successional pathways (*11*).

4. The three successional components will be positively associated as greater woody legacies at land abandonment can act as nuclei for regeneration and contribute to rapid recovery toward structurally developed and diverse forests. Successional pathways are therefore expected to be dominated by trajectories characterized by high start, fast speed, and consistent directionality (i.e., progressive succession), or by low start, slow speed, and weak directionality (i.e., arrested succession).

To illustrate the framework and address these questions, we established 122 permanent plots in recently abandoned agricultural fields across six landscapes and monitored them annually for up to five consecutive years between 2020 and 2025, yielding 478 plot-census combinations (Fig. 1). To ensure comparability across sites, we used standardized protocols for plot establishment and measurement. Plots were 25 m by 25 m (0.0625 ha) and were established mostly at land abandonment (stand age = 0). All trees and shrubs with a stem diameter ≥1 cm were identified to species, tagged, and measured annually for stem diameter and height. The 1-cm stem diameter threshold ensured inclusion of small individuals that shape early community assembly and long-term successional trajectories.

On the basis of the annual census of woody vegetation, we quantified 12 forest attributes representing four complementary groups central to ecosystem functioning and services: (i) structure [aboveground biomass (AGB), maximum stem diameter, and structural complexity], (ii) diversity (species richness and exponentiated Shannon diversity) and successional guild (proportion of late-successional individuals), (iii) functional composition [community-weighted mean (CWM), wood density (WD), leaf mass per area (LMA), and proportion of resprouting individuals], and (iv) potential symbioses [proportion of biotically dispersed individuals, nitrogen fixers, or individuals forming arbuscular mycorrhizal (AM) associations] (*21*, *28*–*33*).

**Fig. 2.**
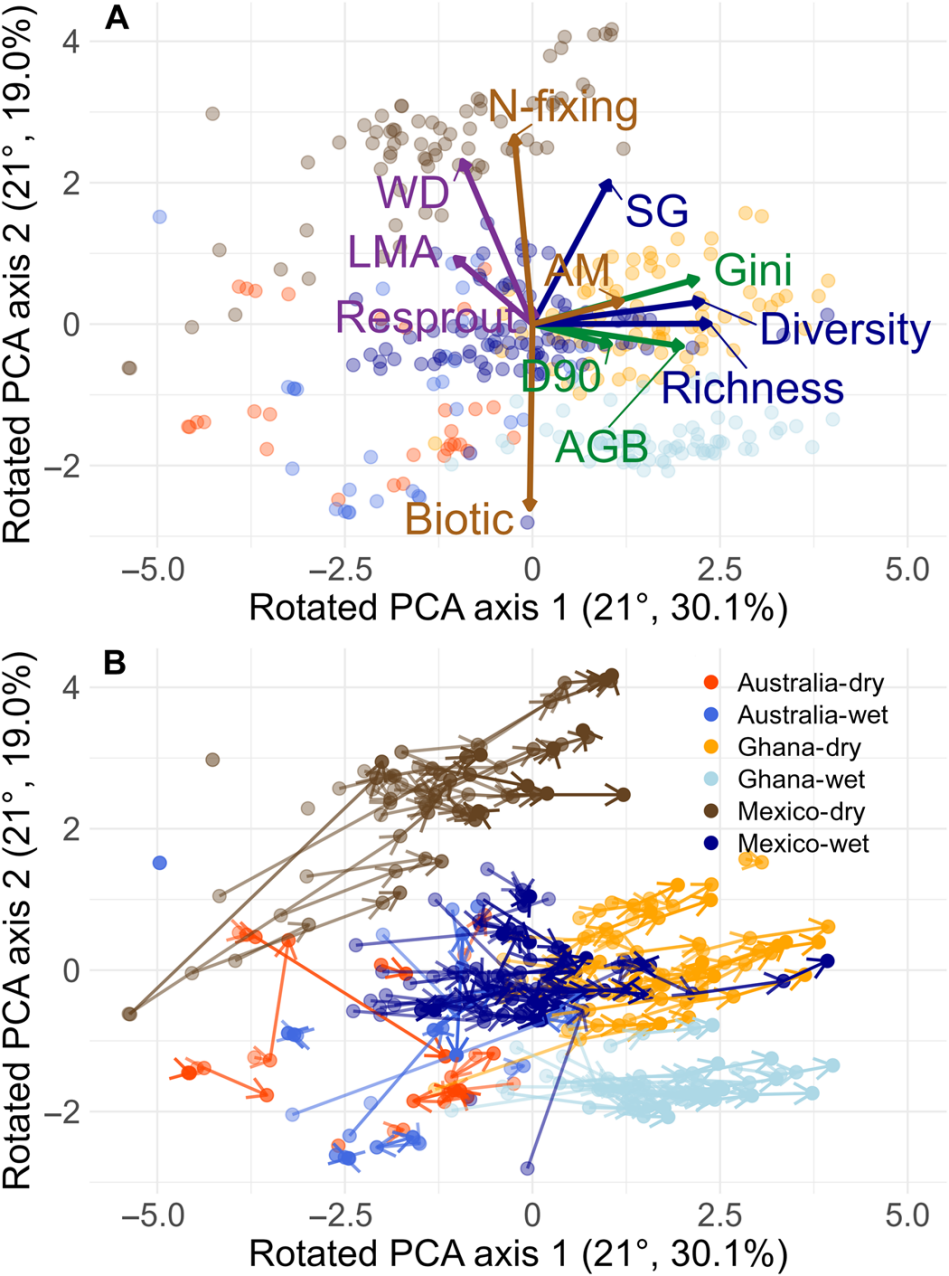
Multivariate forest attribute space in young tropical forests. (**A**) Principal components (PCs) analysis (PCA) of 12 forest attributes across countries and forest types. Colored arrows indicate attribute groups: structure (green), diversity and successional guild (dark blue), functional composition (purple), and potential symbioses (brown). (**B**) Successional trajectories in 2D PCA space, with arrows indicating the direction (the angle) and speed (the length) of change over multiple censuses for each plot. Attribute abbreviations: aboveground biomass (AGB), maximum diameter (D90), Gini coefficient of stem basal area (Gini), successional guild (SG), CWM wood density (WD), CWM leaf mass per area (LMA), proportion of resprouting individuals (Resprout), nitrogen-fixing individuals (N-fixing), biotically dispersed individuals (Biotic), and individuals forming AM associations. PCA axes were rotated to align structure and diversity with PC1.

**Fig. 3.**
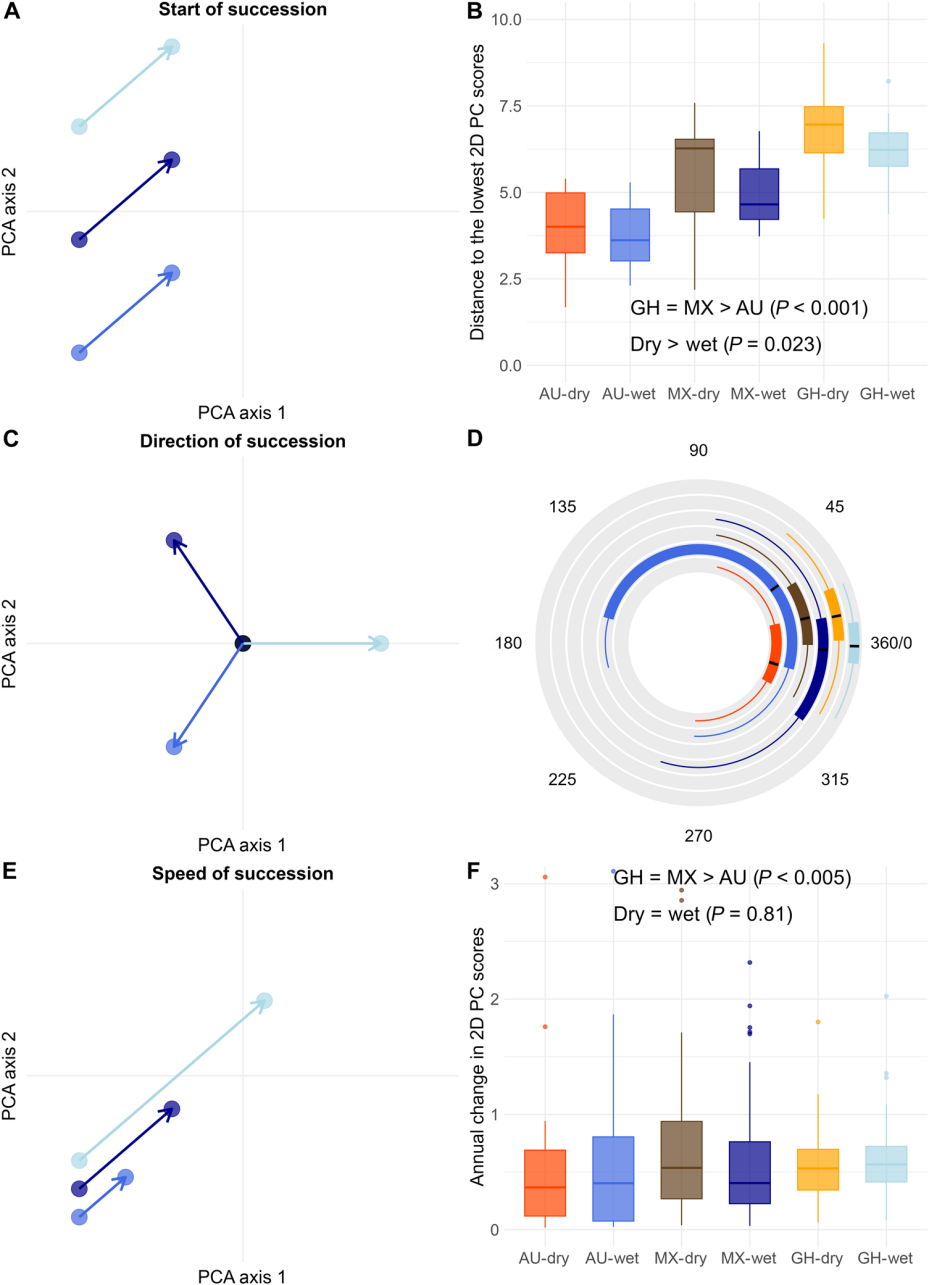
Conceptual representation and empirical patterns of three components of forest succession across countries and forest types. (**A** and **B**) Start of succession, measured as the Euclidean distance from the initial position of each plot to the minimum point in a 2D PCA space. (**C** and **D**) Direction of succession, quantified as the angle of the vector connecting a plot’s position in 2D PCA space at census *t* to its position at census *t* + 1, measured relative to the first PCA axis. (**E** and **F**) Speed of succession, calculated as the Euclidean distance in PCA space between two consecutive censuses of the same plot, divided by the time interval between censuses. In (B), (D), and (F), boxes show the interquartile range (25th to 75th percentile), horizontal lines indicate the median, and whiskers extend to 1.5× the interquartile range; points outside the whiskers represent outliers. Country abbreviations: AU, Australia; MX, Mexico; GH, Ghana. Statistical details are provided in tables S1 to S4.

To identify major axes of forest variation, we performed principal components (PCs) analysis (PCA) using the 12 forest attributes (Fig. 2A). The resulting two-dimensional (2D) PCA space was then used to quantify the three components of succession (Fig. 3). The start was quantified as the Euclidean distance between the initial position of each plot and the lowest observed point (PC1 = −5.4, PC2 = −2.8), capturing variation in initial forest structure, diversity, and functional composition (Fig. 3A). The direction was quantified as the angular trajectory in PCA space (in °, originating in PC1 = 0) between two consecutive censuses, reflecting the orientation of multivariate change over time (Fig. 3C). The speed of succession was quantified as the Euclidean distance in PCA space between two consecutive censuses, divided by the time interval between censuses to obtain an annual rate of multivariate change (Fig. 3E).

To assess how the three components varied across countries and between forest types, we used linear models with the start as the response variable and country and forest type as fixed effects, including stand age at the first census as a covariate. Differences in direction among countries and between forest types were tested using the Watson-Wheeler test for circular data, and within-site directional consistency was assessed using Rayleigh’s test for circular uniformity. Variation in speed was analyzed using linear mixed-effects models with country, forest type, and stand age as fixed effects and plot as a random factor to account for repeated measurements.

Last, we examined relationships among start, speed, and direction in two ways. Before the analysis, each metric was rescaled to a 0-to-100 scale by subtracting the minimum value, dividing by the observed range, and multiplying by 100. First, we used pairwise Spearman’s rank correlations to test bivariate associations among the three components. Second, we evaluated whether successional dynamics were organized around dominant pathways by classifying each rescaled metric as high (≥50%) or low (<50%). Combining these classifications yielded eight possible pathways in 3D successional space. To assess whether some pathways were more dominant than others, we evaluated their frequency relative to the expectation based on equal probability (i.e., each of the eight groups represents 12.5% of the cases).

## RESULTS

### Multidimensional axes of tropical forest succession

The first two PCs (PC1 and PC2) together explained approximately 50% of the total variation in forest structure, diversity, functional composition, and symbiotic associations (Fig. 2). The remaining variation was distributed across higher-order axes (PC3 to PC12), reflecting the inherently multidimensional nature of ecological data. PC1 represented a gradient in forest structure and diversity, ranging from structurally simple, species-poor forests (left) to structurally complex, species-rich forests (right). PC2 captured a gradient in community composition, ranging from forests dominated by early successional, biotically dispersed species (bottom) to those dominated by later-successional species with conservative trait values (e.g., high WD and high LMA) and nitrogen-fixing ability (top).

### Start, direction, and speed of succession

The start of succession, quantified as the Euclidean distance between the initial position of each plot and the lowest observed point, was significantly greater in Ghana and Mexico than in Australia, and greater in dry forests than in wet forests (Fig. 3B and table S1). These differences indicate that plots in Ghana and Mexico, particularly in dry forests, started succession with a more developed structure and diversity and/or with a greater relative abundance of later-successional species with more conservative trait values.

The direction of succession, quantified as the angular trajectory in PCA space, was generally aligned with PC1 (angles near 0°) across all sites except for wet forests in Australia (Fig. 3D). This indicates that early successional change was mainly driven by increases in forest structure and species diversity, with comparatively smaller shifts in functional composition and potential symbiotic associations. Directionality within sites was relatively consistent in all sites except for wet forests in Australia (tables S2 and S3).

The speed of succession, quantified as the Euclidean distance in PCA space between two consecutive censuses, was faster in Ghana and Mexico than in Australia and did not differ significantly between dry and wet forests (Fig. 3F and table S4). The speed was highest immediately following land abandonment and declined over time (table S4), supporting the view that succession is most dynamic in its earliest stages.

### Interrelationships among the three components and dominant successional pathways

**Fig. 4.**
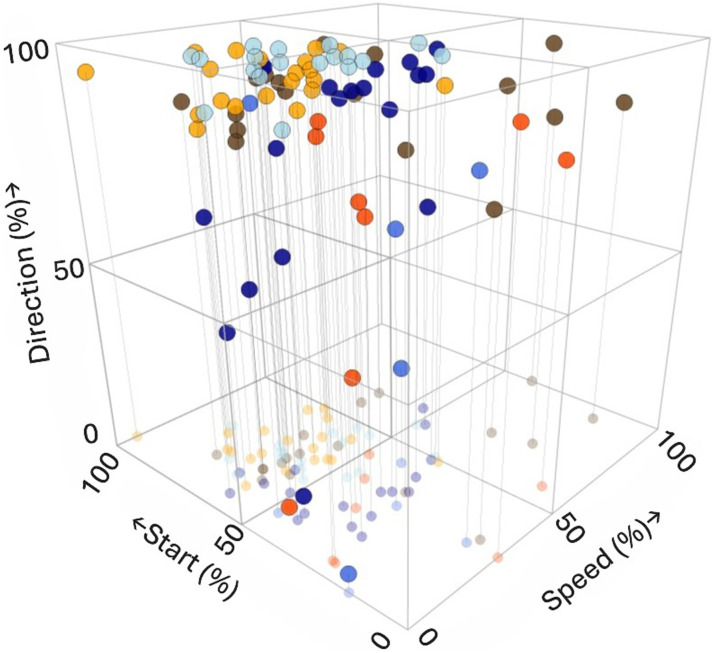
3D relationships among the three components of succession. The start was quantified as the Euclidean distance between the initial position of each plot and the minimum point in the 2D PCA space. The direction was quantified as the angle of the vector connecting a plot’s position in 2D PCA space at census *t* to its position at census *t* + 1, measured relative to the first PCA axis. The speed was quantified as the Euclidean distance between two consecutive censuses of the same plot, divided by the time interval between censuses. Each dot represents one plot (*N* = 87), colored by country × forest type (Australia dry, dark orange; Australia wet, blue; Ghana dry, light orange; Ghana wet, light blue; Mexico dry, brown; and Mexico wet, dark blue). All three components were scaled to a 0 to 100% across all plots. The 3D space is divided into eight cubes corresponding to threshold combinations (≥50 versus <50%) of start, speed, and direction. Vertical lines and shaded projections on the ground plane indicate the position of each point in 2D space defined by start and speed. The count and percentages of plots in each cube are shown in table S5.

During the first 5 years of succession, start, speed, and direction were not significantly associated (fig. S1). When considered jointly in 3D space (Fig. 4), high start (59% of plots), low speed (87%), and high directionality (93%) were most common during early succession (table S5). Consequently, more than half of the plots (54%) clustered within a single pathway characterized by high start, low speed, and high directionality.

## DISCUSSION

We presented a unifying mechanistic framework that captures the three core components of succession and tested it using plots in six tropical landscapes. The start of succession generally declined with the land-use intensity of the countries and was greater in dry than in wet forests. The speed of succession was slowest in the country with the most intensive agricultural land use and was similar between forest types. In general, the direction of succession showed an increase in structural development and species accumulation over time. Last, the start, speed, and direction of early forest succession were largely independent of each other.

### Start of succession

The start of succession differed markedly across countries and between forest types (Fig. 3B). In Ghana and Mexico, where agricultural use was generally shorter and less mechanized, remnant woody vegetation was frequently present at land abandonment (Fig. 1), resulting in higher initial values for forest structure, diversity, and functional composition (*19*, *20*, *34*). In contrast, in Australia, lands had been used more intensively for a longer period (in most cases exceeding 50 years). Woody remnants are therefore rare, and plots were frequently dominated by tall non-native grasses and ferns (Fig. 1), resulting in lower starting values (*35*–*37*).

Dry forests started succession with communities more strongly dominated by later-successional species with resource-conservative trait values than wet forests (Fig. 3B), possibly due to two mechanisms. First, dry forests often exhibit abundant resprouting from roots and stumps because resprouting is a common regeneration strategy under recurrent disturbances such as fire and drought (*38*, *39*). These resprouting individuals can contribute to the immediate recovery of species composition after land abandonment (*40*). Second, seasonally dry climates impose strong environmental filters, favoring species with tough leaves and dense wood that enhance drought tolerance (*41*–*43*) as well as those with nitrogen-fixing ability that improves water-use efficiency (*29*, *44*). In contrast, wet forests started with communities more strongly dominated by fast-growing pioneer species with more acquisitive trait values (soft leaves and low-density wood), which promote rapid growth in benign environments (*45*, *46*). Wet forest communities also harbored a high proportion of biotically dispersed species, reflecting stronger plant-animal coevolution in climatically stable environments (*47*).

In sum, low previous land-use intensity resulted in more biological legacies and more developed and diverse communities at the start of succession. Environmental filtering led to a different start of succession in dry and wet forests; dry forests started with drought-tolerant conservative species, and wet forests with fast-growing acquisitive animal-dispersed species (*43*).

### Direction of succession

Across all sites, succession mainly progressed along PC1 (Fig. 3D). This indicates that early successional development is characterized by structural development and species accumulation rather than by major shifts in functional composition or symbiotic associations.

We hypothesized that the direction of succession would be more variable at intermediate land-use intensity (as in Mexico) (*13*). Instead, we found that, within sites, successional trajectories were relatively consistent, except for wet forests in Australia (table S2). The greater variability in this site may reflect heterogeneity in previous land-use intensity and duration, leading some plots to progress toward increased structure and diversity (angles near 0°), while others experienced arrested succession due to stronger competition from grasses and ferns (Fig. 1, angles near 180°) (*26*, *27*). In addition, the low number of recruits in Australian plots may result in more stochastic changes in functional and symbiotic composition (i.e., along PC2) as these changes depend strongly on the species identity of new recruits. Combined, these factors may contribute to the greater variation in successional trajectories observed on this site.

In sum, the first 5 years of secondary succession are generally directional and predictable (*3*, *17*, *32*, *48*). However, spatial heterogeneity in human land use, together with stochastic variation in species arrival and colonization, can generate variability in successional direction and reduce predictability (*10*, *13*, *16*, *19*).

### Speed of succession

The speed of succession was fastest in Ghana and Mexico (Fig. 3E) possibly because higher abundances of resprouting individuals and saplings at land abandonment accelerated early successional recovery (Fig. 1). In both countries, woody saplings often regenerated during the cropping phase, and manual clearing with a machete resulted in more resprouting individuals (*20*, *34*). In contrast, prolonged and mechanized pasture use in Australia was associated with reduced woody legacies and strong dominance of grasses and ferns (Fig. 1). Such high dominance of herbaceous species intensifies space and resource competition, suppresses tree establishment, and slows down or temporarily arrests succession (*35*, *36*).

Unexpectedly, the speed of succession did not differ significantly between dry and wet forests possibly because we focused on the first 5 years of succession, in which recovery may be more strongly influenced by biological legacies such as the abundance of resprouting individuals, and competing growth forms (e.g., grass and ferns) (Fig. 1) than by forest type per se (*11*, *49*). Resprouting is often more common in drier ecosystems (*38*, *39*) and may promote rapid early recovery in dry forests (*50*, *51*), while higher abundance of competing growth forms in wetter ecosystems may hinder initial forest recovery (*26*, *27*, *52*). As succession advances, however, the relative importance of these biological legacies may diminish, while environmental conditions that determine the length and conditions of the growing season may become more important. This shift could lead to the commonly observed pattern of faster recovery in wet than in dry forests over longer timescales (*22*, *23*).

In sum, lower previous land-use intensity was associated with higher abundance of resprouts and saplings and faster successional speed, while dry and wet forests differ notably in ecology but not in early successional speed. Early recovery dynamics are therefore primarily driven by the social rather than the ecological component of the socioecological system.

### A unified mechanistic framework of secondary succession

Our conceptual framework integrates three key components of succession (start, speed, and direction). By monitoring multiple forest attributes and analyzing them in a multivariate space, we can (i) quantify the three successional components simultaneously, (ii) disentangle how successional trajectories vary across landscapes, and (iii) identify dominant successional pathways. The start is an important but often overlooked component of succession because the type, quantity, and quality of biological legacies fundamentally distinguish secondary succession from primary succession (*11*). It can also explain the large variation in the pathways of secondary succession (*10*, *53*). Furthermore, our framework conceptually disentangles speed and direction in a clear way, and it can also be applied for the analysis of individual attributes over time using regression analysis (i.e., in a univariate context), where the intercept represents the start, the sign of the slope represents the direction, and the magnitude of the slope represents the speed of succession.

We hypothesized that the three components of succession would be positively associated as a high start would also lead to a fast speed and directionality in succession. However, we found no significant associations among them during the first years of succession (fig. S1). This independence among the three successional components may also explain the large variation observed in successional pathways within and across sites (*54*). When longer successional timescales are considered, the direction and speed of succession may become more tightly coupled as forest attributes converge toward old-growth forest conditions (*3*). Alternatively, they may remain decoupled because of ecological feedback (e.g., resource competition and facilitation) that drives alternative successional pathways (*10*, *15*).

Applying our framework to young secondary tropical forests, we found that of all eight possible combinations of start, speed, and direction, more than half of all plots (54%) clustered within a single dominant pathway characterized by high starting conditions, low recovery speed, and strong directionality (table S5). This dominant pathway suggests that early secondary succession on abandoned agricultural fields typically starts with woody legacies but progresses slowly probably because it takes time to overcome the first barriers for regeneration, such as source and dispersal limitation in fragmented landscapes (*55*), and establishment limitation in harsh early successional conditions or because of competing growth forms (*27*). Once sufficient trees are established, they can act as nuclei of regeneration (*56*) and fuel succession because they out-shade competing grasses and ferns (*57*) and attract frugivores, thus increasing the local seed rain. In combination, this may lead to a predictable direction of succession toward structurally complex and diverse forests.

### Implications for restoration

Our proposed framework provides a tool to identify priority areas for different tropical forest restoration methods. By applying this framework to time-series analyses of high-resolution remote-sensing imagery or large monitoring networks of secondary forests (e.g., 2ndFOR), the start, speed, and direction of succession can be quantified across broad spatial scales (*55*). Such analyses can help guide restoration strategies and interventions based on combinations of successional components (table S6).

When succession is fast and strongly directional toward structurally complex and biodiverse forests, natural regeneration can be used as a scalable and low-cost restoration approach, regardless of the start (*58*, *59*). When succession is either slow or weakly directional, assisted natural regeneration may be more appropriate (table S6). In sites with high start but weak directionality, succession may be constrained by the dominance of invasive or highly competitive early-successional woody species (e.g., *Lantana camara*) that inhibit further recovery. In such cases, thinning or gap creation can trigger understory reinitiation by releasing suppressed saplings and creating opportunities for new woody plants to establish (*60*, *61*). In sites with low start and weak directionality, succession can be redirected by enrichment planting of fast-growing, fleshy-fruited early-successional species that create perching sites for birds and enhance seed dispersal (*24*, *62*). When successional speed is slow, the main limiting factor (e.g., dispersal, establishment, or growth) should be identified to design targeted interventions. Dispersal limitation can be overcome by direct seeding or enrichment planting of early- and late-successional species in sites with low start (*63*, *64*), or by protecting and liberating resprouts and saplings in sites with high start (*24*, *60*). Establishment and growth can be promoted by improving soil water and nutrient availability through soil scarification or amendments and by reducing competition for space and resources through the removal of competing growth forms such as grasses or ferns (*61*, *65*, *66*).

Last, when succession is both slow and weakly directional, regardless of the start, multispecies tree planting using combinations of early- and late-successional species may be needed to initiate recovery and steer succession toward structurally complex and biodiverse forests (*67*, *68*). When active tree planting is required, species should be selected that not only provide social and economic benefits to local people (*69*, *70*) but also have the right traits to survive and grow under local environmental conditions. In dry forests, species with high WD and nitrogen-fixing ability may be more successful as these traits confer tolerance to drought and heat stress (Fig. 2A). In wet forests, planting biotically dispersed species can enhance habitat quality and food resources for frugivores (Fig. 2A), thereby facilitating seed input from surrounding forests and accelerating succession (*68*).

In sum, we advanced successional theory by analyzing succession in terms of its three underlying components. This provides a more comprehensive and mechanistic understanding of succession and offers insight into where, when, and how natural regeneration can successfully be used as a nature-based solution for forest restoration.

## MATERIALS AND METHODS

### Study system

We focused on secondary tropical forest succession following agricultural land abandonment as pasture and shifting cultivation are the predominant drivers of tropical deforestation worldwide (*71*) and the main areas where forest regrowth occurs (*5*, *59*). We focused on early succession right after land abandonment because (i) it is the phase when the largest successional changes occur (*28*, *33*), and (ii) it determines future successional pathways and therefore the extent to which forests can recover (*9*, *15*). Most successional studies ignore this very early phase, and either start after several years when woody vegetation has already been established or only measure larger trees (>5-cm stem diameter) (*33*), and not the regenerating trees (≥1-cm stem diameter) that determine the future composition of the forest canopy.

### Study sites

Research was conducted in three countries (Mexico, Ghana, and Australia), each in a different continent (North America, Africa, and Oceania). This pantropical design enabled us to identify general patterns and context-specific differences in succession. These three countries differ not only in their biogeographic conditions, with distinct floristic composition, but also in their levels of socioeconomic development (Australia > Mexico > Ghana), which influence land tenure, management practices, and land-use intensity (Fig. 1) (*72*, *73*) and, hence, succession. Within each country, we selected two forest types (dry and wet) that represent the two main forest types in the lowland tropics, with potentially large differences in ecosystem functioning and succession (*22*, *23*). An overview of all site locations and characteristics is presented in Fig. 1.

### Plot establishment and vegetation measurements

A total of 122 permanent plots (25 m by 25 m; 0.0625 ha) were established in recently abandoned pastures or agricultural lands at 0 to 2 years since land abandonment. Plot size (25 m by 25 m) was chosen to accommodate the common practice of shifting cultivation in the study landscapes, which often occurs in relatively small lands (often ∼30 m by 30 m, occasionally up to 0.5 ha). Most plots had been completely cleared before agricultural use, although remnant trees were occasionally retained for shade, fruit production, or cultural reasons, or left standing because of limited mechanization (e.g., in Ghana) (*34*, *74*). Plots were spaced at least 50 m apart to assure sufficient spatial independence. Within each site, plots were selected from a single dominant agricultural land-use type (except for the Mexican dry forest site, which was a mix of crop fields and pasture) to minimize within-site variation in previous land-use type (Fig. 1). Plots located in pasture lands were fenced where necessary to exclude cattle and prevent browsing, thereby allowing natural forest regeneration to proceed. Without fencing, continued grazing would have maintained the sites in an active pasture state. All trees and shrubs with a stem diameter ≥1 cm [measured at 30 cm aboveground in Mexican dry forests and at breast height (1.3 m) in all other sites] were identified, tagged, and measured annually for up to five consecutive years, resulting in 478 plot–census combinations. The relatively low diameter threshold (≥1 cm) was adopted to capture early recruitment dynamics that drive changes in structure, diversity, and functional composition during the initial stages of succession (*75*).

### Multidimensional forest recovery

We quantified 12 forest attributes from four complementary groups: (i) forest structure, (ii) species diversity and successional guild, (iii) functional composition, and (iv) potential symbioses. These different attributes are key aspects of ecosystem functioning: Forest structure influences biomass accumulation and habitat complexity (*30*, *76*), species diversity underpins ecosystem functioning and resilience (*77*, *78*), functional composition reflects resource use strategies and productivity (*79*, *80*), and potential symbioses facilitate seed dispersal and nutrient cycling (*81*–*83*). To provide a coherent and balanced overview, we used three attributes per group. All attributes were derived from standardized measurements of aboveground vegetation.

### Forest structure

We described forest structure using AGB, the size of the largest trees, and heterogeneity in forest structure.

#### Aboveground biomass

AGB (in megagrams per hectare) was estimated using the pantropical tree biomass allometric equation, using stem diameter, species-specific stem WD (in grams per cubic centimeter), and a measure of environmental stress (E) (*84*). WD data were obtained from local site measurements, complemented with the global database (*30*, *40*, *85*–*90*). When WD was unavailable for some species, we used the highest taxonomic resolution available (genus or family level) or the site-average WD. E is a composite variable derived from climatic factors, such as temperature seasonality, precipitation, and climatic water deficit, and is used to adjust the tapering factor to estimate tree height from diameter at breast height (DBH) under different environmental conditions (*84*). Although this equation was developed for trees ≥5 cm DBH and may overestimate biomass for smaller individuals (1 to 5 cm DBH), we applied a single pantropical equation across all sites to ensure methodological consistency and comparability instead of using different site- or region-specific allometric equations for secondary forests. Plot-level AGB was calculated by summing individual tree biomass and scaling to a per-hectare basis.

#### *Maximum tree size* (*D90*)

D90 (in centimeters) is defined as the 90th percentile of stem diameter within each plot. Large trees have high conservation value because they provide habitat, shelter, and food resources for epiphytes, lianas, fungi, animals, and insects, and contribute disproportionately to carbon storage (*91*, *92*). Using the 90th percentile reduces sensitivity to the plot size and single remnant outliers while still capturing the upper structural limit of the stand.

#### Structural heterogeneity

Structural complexity (dimensionless) captures the variation in tree sizes within a plot, reflecting vertical layering and forest maturity. We quantified structural complexity using the unweighted Gini coefficient of individual stem basal area, calculated with the “gini.wtd” function in the dineq R package (*93*). The Gini coefficient ranges from 0 (uniform stem basal area) to 1 (maximum variation). It was computed as the sum of all absolute differences in stem basal area across all pairwise combinations of the number of trees (*N*) in the plot, divided by 2 × *N*^2^ × mean stem basal area of all trees.

### Species diversity and successional guild

We described species diversity based on the two measures of species diversity using Hill numbers and the successional guild, based on the proportion of later successional species.

#### Species richness and diversity

We quantified species diversity using two Hill numbers. Species richness was calculated as the Hill number of order zero (^0^*D*) (*94*), which assigns equal weight to all species regardless of their abundance within a plot. Exponentiated Shannon diversity [Hill number of order one; ^1^D = exp (H′)] was calculated to represent the effective number of common species required to produce the observed diversity (*94*). Although species diversity can be sensitive to sampling completeness in species-rich tropical systems (*95*), we did not standardize Hill numbers on the basis of sampling coverage for all analyses for two reasons. First, sampling effort was standardized across all plots through identical plot size and inclusion criteria. Second, because the study focuses on early stages of succession following land abandonment, many plots at the initial census contain only one or a few individuals. This results in a high proportion of singleton and doubleton species and, consequently, low sample coverage, making coverage-based standardization less practical.

#### Successional guild

In this study, successional guild was defined as the proportion of late-successional individuals in a plot, providing an indicator of its compositional similarity to old-growth forests. For each country, species’ successional status was derived from published studies (*21*, *87*, *96*, *97*). Species were classified as early-, mid-, or late-successional based on temporal dynamics, persistence during succession, and light requirements for growth and survival. Early-successional species are predominantly light-demanding, recruiting within the first 5 years of succession (<5 years) (*98*) and dominating for the first 20 years (*99*). Mid-successional species establish and grow under the canopy of early-successional species, typically dominating succession between 20 and 100 years (*99*). Late-successional species are shade-tolerant, recruit continuously throughout succession, and dominate the canopy after 100 years until major disturbances reset succession (*99*). To calculate the successional guild, we assigned numerical values to species (early = 0, mid = 0.5, and late = 1.0) and weighted them by their relative abundances in each plot. We weighted it by the relative abundance rather than basal area because stem number better reflects community composition of species that can attain different sizes and because basal area is strongly influenced by remnant trees, especially in the first years of succession. Species without successional status information were excluded from the calculation. Plot-level values were expressed as proportions, with higher values indicating a larger proportion of late-successional individuals.

### Functional composition and potential symbioses

Functional trait composition and potential symbioses of the community were described using the CWM values of functional traits (stem WD, LMA, and resprouting) and potential symbioses (seed dispersal mode, nitrogen-fixing ability, and association with AM fungi). CWMs represent the average trait value in a community weighted by species abundances and are closely linked to community assembly, ecosystem processes, and functioning (*30*, *80*, *100*). CWM trait values were calculated by multiplying each species’ trait value by its relative abundances within the plot and then summing across all species. CWM trait values were calculated using species’ relative abundance rather than relative dominance (e.g., basal area or AGB). This approach was chosen (i) to reduce the disproportionate influence of large remnant trees and (ii) because abundance-weighted CWMs more directly reflect community reassembly processes during early succession. Species without trait data were excluded from the calculation of CWM. Trait data were collected locally at each study site to account for local environmental conditions (*30*, *40*, *85*, *87*, *88*, *101*–*105*). Stem WD and LMA were primarily measured on sun-exposed individuals between 1 and 8 m in height, which corresponds to the typical size range in early successional stands. When necessary, these measurements were supplemented with species-level values from global trait databases (*89*, *90*). Species mean values were used for the analysis, and thus, intraspecific plasticity related to individual size, light environment, or successional stage was not incorporated, and changes in CWM trait values therefore reflect changes in species abundance or species turnover, rather than within-species acclimation. To ensure robust estimates, we excluded plots for which the trait values for less than 75% of the individuals were known (46 of 478 plot-census combinations).

#### Wood density

Stem WD (in grams per cubic centimeter) is the wood dry mass divided by the wood fresh volume. WD is important for carbon cycling because low WD increases stem hydraulic conductivity, photosynthetic carbon gain, and volumetric growth capacity, but decreases tissue density, whereas high WD is associated with longer-lived tissues and increased carbon residence time (*106*, *107*).

#### Leaf mass per area

LMA (in grams per square centimeter) is the ratio of leaf mass to leaf area and reflects the cost of leaf construction. LMA is a key component of the leaf economics spectrum and scales positively with leaf longevity but negatively with photosynthetic capacity (*46*). Hence, it is related to the forest productivity (*79*).

#### Resprouting

Resprouting is the ability to regenerate shoots or stems from existing structures (e.g., stumps, roots, or branches) after disturbance or damage (*38*). This strategy is particularly common in frequently disturbed or dry environments (*108*, *109*), enabling rapid recovery after major disturbances or stem dieback due to drought. In the field, each individual was visually assessed as originating from seed or resprouting, and the proportion of resprouted individuals was calculated for each plot (*100*).

#### Biotic seed dispersal

Dispersal mode refers to the vector by which seeds are transported from the parent plant to new locations, playing a critical role in the recovery of species diversity and composition in naturally regenerating forests (*12*, *55*). Information on the dispersal mode (e.g., birds, bats, mammals, wind, water, and gravity) for each species was collected from literature and books (*21*, *67*, *87*, *110*, *111*). Species were subsequently classified as either biotically dispersed (coded as 1) or abiotically dispersed (coded as 0).

#### Nitrogen fixation

Biological nitrogen (N) fixation is the result of a symbiotic relationship between certain tree species and *Rhizobium* bacteria. It provides the major nitrogen input to tropical forests and is an important source of nitrogen recovery during secondary tropical forest succession on abandoned agricultural fields (*14*, *29*). We extracted information on whether tree species fix nitrogen from available databases (*112*). Because actual N fixation rates depend on *Rhizobium* activity, which can be affected by local conditions, we assessed potential rather than realized fixation. Species were classified as nitrogen fixing (i) or non–nitrogen fixing (0).

#### AM association

AM associations are symbiotic relationships between *Glomeromycota* fungi and plant roots, facilitating the exchange of nutrients, especially phosphorus, in exchange for carbon from the plant. This mutualistic interaction is crucial for water and nutrient uptake in tropical forests, especially for immobile nutrients such as phosphorus. AM plays, therefore, an important role in nutrient cycling. We extracted information on whether tree species form AM associations from available databases (*113*, *114*). As with N fixation, we only considered species-level potential, not realized mycorrhizal colonization. Species were classified as AM (1) or non-AM (0).

### Statistical analysis

To quantify the three components of succession, namely start, speed, and direction, we performed a PCA using the “prcomp” function in R, using 12 standardized forest attributes as response variables and 366 plot-census combinations as observations. Each attribute was standardized by subtracting the mean value across all plots and dividing by the SD. PCA revealed two major axes (Fig. 2A): PC1 explained 30.1% of the variation and was primarily associated with structural and diversity attributes, and PC2 explained 19.0% of the variation and captured differences in functional composition and potential symbiotic associations. Axes were rotated by −21° to align structure/diversity with PC1, facilitating visualization and interpretation of successional trajectories. Individual structural, diversity, functional, and symbiotic attributes may nevertheless deviate from these integrated patterns, and therefore, future studies could analyze such attribute-specific trajectories, which will be important for understanding how different dimensions of recovery unfold through time.

The start of succession was quantified as the Euclidean distance between the initial position of each plot and the lowest bottom-left point observed in PCA space (PC1 = −5.4, PC2 = −2.8; Fig. 2A), thus capturing variation in initial structure, diversity, and functional composition at land abandonment (Fig. 3A). Larger distances indicate more structurally, taxonomically, and functionally advanced conditions at the start of succession. This reference point was chosen because PC1 spans a gradient in structure and diversity gradient (from simple, species-poor at the left to complex, species-rich forests at the right), while PC2 distinguishes communities dominated by early-successional pioneers with acquisitive trait values (e.g., low WD and low LMA) at the bottom from those dominated by later-successional species with conservative trait values (e.g., high WD and high LMA) at the top. Accordingly, higher PC1 scores indicate greater structural complexity and diversity, whereas higher PC2 scores indicate stronger abundance of later-successional, conservative species. To test how the start of succession varied across countries and forest type, we fitted a linear model with Euclidean distance as the response variable, and country and forest type as fixed effects (*N* = 90 plots). We also included stand age at the initial census as a covariate to account for potential age effects. Post hoc tests were performed using the “emmeans” package (*115*), with *P* values adjusted using the Tukey method.

The direction of succession was quantified as the angle of the vector connecting a plot’s position in 2D PCA space at census *t* to its position at census *t* + 1, measured relative to the first PCA axis (Fig. 3C). This angle reflects the orientation of multivariate change over time. To assess whether successional directions were consistent within sites, we applied Rayleigh’s test for circular uniformity (*116*, *117*), which assesses whether angles deviate from random expectation. We further compared successional directions across sites using the Watson-Wheeler test based on the median directional angle per plot over the consecutive census intervals (*N* = 94 plots) (*118*). Median directional angles per plot were used for both analyses as these circular tests cannot accommodate random effects or repeated measures. Post hoc tests were performing the emmeans package (*115*), with *P* values adjusted using the Benjamini-Hochberg procedure to control for the false discovery rate (i.e., the expected proportion of significant results that are false positives).

The speed of succession was quantified as the Euclidean distance in 2D PCA space between two consecutive censuses of the same plot, divided by the time interval between censuses to obtain an annual rate of multivariate change (Fig. 3E). We fitted a linear mixed-effects model using the “lmer” function from the lme4 package (*119*). In the model, country, forest type, and stand age were included as fixed effects (*N* = 264 plot-census combinations), and plot was included as a random effect to account for repeated measurements. Post hoc comparisons were conducted using the emmeans package (*115*), with *P* values adjusted by the Bonferroni method for robust, conservative inference for main effects.

Last, to assess relationships among start, speed, and direction, we rescaled each metric to a 0-to-100 scale by subtracting the minimum value, dividing by the observed range, and multiplying by 100. Associations among the three components were examined using pairwise bivariate relationships with Spearman’s rank correlation tests (fig. S1) and by visualizing their joint distribution in 3D space (Fig. 4). For speed and direction, we used plot-level medians across census intervals. Given the circular nature of directional data and the tendency for trajectories to align with PC1 (0°), we expressed rescaled direction as the absolute angular deviation from PC1, rescaled to 0 to 100. Higher values (closer to 100) indicate trajectories aligned with PC1, reflecting progressive development of structure and diversity, whereas lower values (closer to 0) indicate regressive trajectories.

To assess whether succession is structured around dominant pathways, we classified each rescaled metric as high (≥50%) or low (<50%) and combined them in 3D space, yielding eight possible combinations of start, speed, and direction. To assess whether some pathways were more dominant than others, we evaluated their frequency relative to the expectation based on equal probability (i.e., each of the eight groups represents 12.5% of the cases) (table S5). In plots without woody individuals, certain attributes could not be calculated. These plots were therefore excluded, reducing the dataset to 87 plots for this analysis.
