# Supplementary material for: Start, speed, and direction of secondary forest succession
Source: Sci Adv. 2026 Aug 7;12(32):eaed5470. doi: 10.1126/sciadv.aed5470 (PMC13450221; doi:10.1126/sciadv.aed5470)
Supplement: Supplementary file 1 [file sciadv.aed5470_sm.pdf]

Science Advances
AAAS

Supplementary Materials for

Start, speed, and direction of secondary forest succession

Tomonari Matsuo *et al.*

Corresponding author: Tomonari Matsuo, tomonari.matsuo@wur.nl

Sci. Adv. **12**, eaed5470 (2026)
DOI: 10.1126/sciadv.aed5470

This PDF file includes:

Fig. S1
Tables S1 to S6

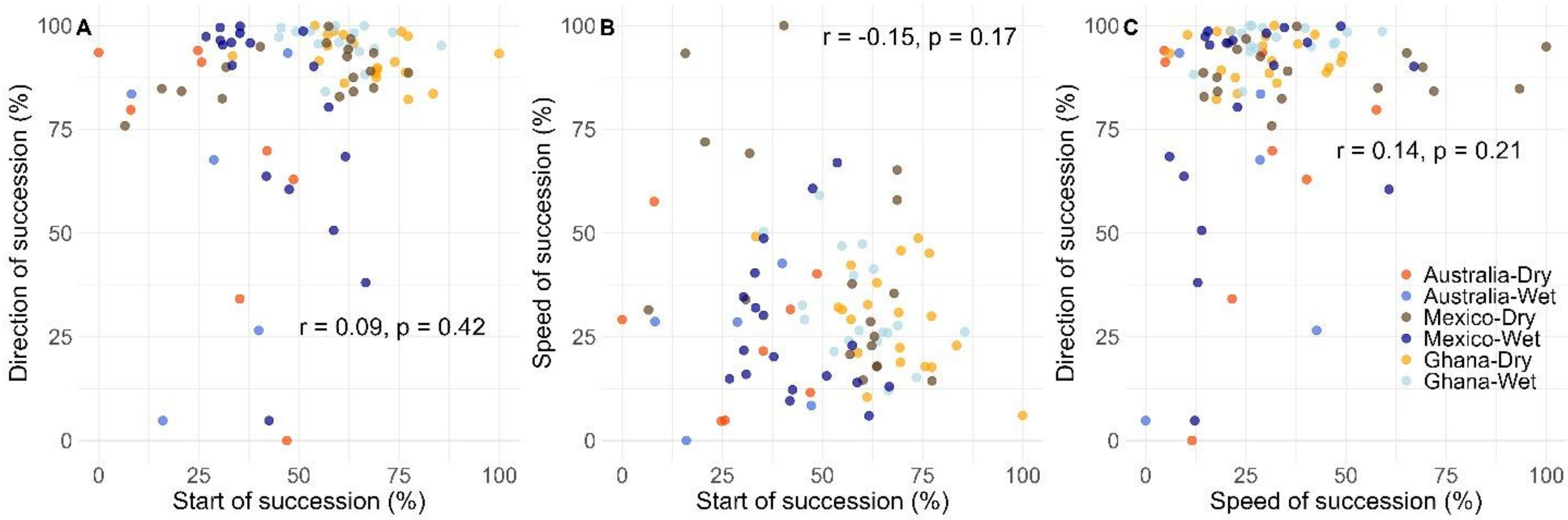

Fig. S1. Pairwise relationships between normalized measures (0–100%) of the start, speed, and direction of succession across all study plots (N=87 plots). Pairwise associations were assessed using Spearman's rank correlation (r). The start was quantified as the distance between the initial position of each plot and the minimum point in the two-dimensional PCA space. The speed was calculated as the Euclidean distance between two consecutive censuses of the same plot, divided by the time interval between censuses. The direction was quantified as the angle of the vector connecting a plot's position in 2D PCA space at census t to its position at census t+1, measured relative to the first PCA axis.

Table S1. Results of the linear model and post hoc pairwise comparisons for the start of succession (N=90 plots).
The start of succession was quantified as the Euclidean distance between the initial position of each plot and the lowest observed point in PCA space (Fig. 3A). The model tested the effects of country, forest type, and stand age at the initial census. Post hoc pairwise comparisons were performed using estimated marginal means (emmeans) with Bonferroni adjustment. Significant effects ($p < 0.05$) are shown in bold.

Effect/Contrast	Degrees of freedom	F value	Estimate	Standard error	p value
**ANOVA**					
Country	2	33.9			**<0.001**
Forest type	1	6.5			**0.022**
Age	1	10.0			**0.0022**
Residuals	85				
**Pairwise Comparisons**					
Australia-Ghana	85		-2.1	0.40	**<0.001**
Australia-Mexico	85		-1.5	0.34	**<0.001**
Ghana-Mexico	85		0.58	0.33	0.18
Dry-Wet	85		0.53	0.23	**0.023**

Table S2. Rayleigh's test for directional consistency (i.e., homogeneity) of successional trajectories within each site. The direction of succession was quantified as the angle of the vector connecting a plot's position in 2D PCA space at census t to its position at census t+1, measured relative to the first PCA axis. This angle reflects the orientation of multivariate compositional change over time (Fig. 3C). Rayleigh's Z statistics and associated p-values are shown in the table. Significant effects ($p < 0.05$) are shown in bold and indicate that the direction is homogeneous. Because Rayleigh's test cannot accommodate random effects or repeated measures, analyses were based on the median directional angle per plot over the consecutive census intervals. Results were consistent when using all directional data instead of plot-level medians.

Site	Sample size	Z statistic	p value
Australia-Dry	10	0.54	**0.048**
Australia-Wet	8	0.28	0.53
Ghana-Dry	20	0.98	**<0.001**
Ghana-Wet	18	0.99	**<0.001**
Mexico-Dry	19	0.92	**<0.001**
Mexico-Wet	19	0.68	**<0.001**

Table S3. Watson–Wheeler tests for successional direction and post hoc pairwise comparisons (N=94 plots). The omnibus test indicates if there are significant differences in angular distributions across countries ($W = 27.0$, $df = 4$, $p < 0.001$) and between forest types ($W = 7.3$, $df = 2$, $p = 0.026$). The degrees of freedom are determined by the number of groups compared ($df = 2 \times$ [number of groups − 1]). Post hoc pairwise comparisons were performed using Watson–Wheeler tests with Benjamini–Hochberg correction for multiple comparisons. Significant effects ($p < 0.05$ after adjustment) are shown in bold. Because the Watson–Wheeler test cannot accommodate random effects or repeated measures, analyses were based on the median directional angle per plot over the consecutive census intervals.

Effect/Contrast	W	p-value
Australia – Ghana	22.8	**<0.001**
Australia – Mexico	7.7	**0.021**
Ghana – Mexico	9.7	**0.012**
Dry – Wet	7.3	**0.026**

Table S4. Results of the linear mixed-effects model (LMM) and post hoc pairwise comparisons for the speed of succession (N=264 plot-census combinations). The speed of succession was quantified as the Euclidean distance in 2D PCA space between two consecutive censuses of the same plot, divided by the time interval between censuses to obtain an annual rate of multivariate change (Fig. 3E). The LMM tested the effects of country, forest type, and stand age, with plot included as a random effect. Post hoc pairwise comparisons were performed using estimated marginal means (emmeans) with Bonferroni adjustment. If the estimate is positive, then the first part of the contrast (e.g., Dry) has a faster speed than the second part of the contrast (e.g., Wet). Significant effects ($p < 0.05$) are shown in bold. The speed of succession was $\log_{10}$-transformed prior to analysis to improve the normality of residuals.

Effect/Contrast	Estimate	Standard error	Degrees of freedom	t ratio/value	p-value
Australia – Ghana	–0.34	0.076	108.6	-4.4	**<0.001**
Australia – Mexico	–0.25	0.074	97.9	-3.4	**0.0030**
Ghana – Mexico	0.084	0.056	82.5	1.5	0.41
Dry – Wet	0.012	0.051	85.3	0.24	0.81
Age	-0.14	0.022	224.7	-6.3	**<0.001**

Table S5. Distribution of plots across threshold combinations (≥50%) of normalized measures (0–100%) of the start, direction, and speed of succession across all study plots (N=87 plots). For each group, counts, percentages, and cumulative percentages are shown, ordered from the group with all variables ≥50% to the group with all variables <50%.

Group	N	Percentage	Cumulative percentage
Start ≥50 & Direction ≥50 & Speed ≥50	3	3.5	3.5
Start ≥50 & Direction ≥50 & Speed <50	47	54.0	57.5
Start ≥50 & Direction <50 & Speed ≥50	0	0	57.5
Start <50 & Direction ≥50 & Speed ≥50	8	9.2	66.7
Start ≥50 & Direction <50 & Speed <50	1	1.1	67.8
Start <50 & Direction ≥50 & Speed <50	23	26.4	94.3
Start <50 & Direction <50 & Speed ≥50	0	0	94.3
Start <50 & Direction <50 & Speed <50	5	5.7	100

Table S6. Recommended forest restoration methods (FRMs) and potential management interventions based on the start, speed, and direction of succession. Start, speed, and direction were classified as high (≥50%) or low (<50%). NR = natural regeneration, ANR = assisted natural regeneration (including enrichment planting to assist natural recovery), and TP = tree planting.

Start	Speed	Direction	FRMs	Potential management interventions	Rationale
High/low	High	High	NR	No intervention is needed	Succession is already proceeding rapidly toward structurally complex and biodiverse forests (59, 60)
High	High	Low	ANR	Reduce dominance of invasive or highly competitive early successional woody species through cutting, thinning, or weeding	To release suppressed saplings and create opportunities for new woody plants to establish (61, 62)
Low	High	Low	ANR	Enrichment planting of tall, fast growing fleshy fruited early-successional species	To create high perching sites for birds, improve soil and microclimate, and create nuclei for regeneration (24, 63)
High	Low	High	ANR	Remove grass, ferns, and vines through spraying or mowing to improve tree growth	To protect and liberate saplings and resprouts to increase the survival and growth of existing woody vegetation (62, 66)
Low	Low	High	ANR	1. Direct seeding or enrichment planting of early- and late-successional species<br>2. Soil scarification or amendments<br>3. Remove grass, ferns, and vines through spraying or mowing to improve tree growth	1. To promote establishment of diverse tree species (64, 65)<br>2. To enhance tree establishment, survival, and growth by improving soil water/nutrient availability (67)<br>3. To improve survival and growth of newly established trees by reducing competition for space and resources (62, 66)
High/low	Low	Low	TP	1. Multi-species tree planting using a mix of early- and late-successional species (*11*, *12*)<br>2. Wet forests: Prioritize biotically dispersed species<br>3. Dry forests: Prioritize species with high wood density and/or nitrogen-fixing ability	1. To promote establishment of diverse tree species (68, 69)<br>2. To enhance habitat quality and food resources for frugivores, thereby facilitating seed input from surrounding forests (our study)<br>3. Because they can tolerate hot and dry early-successional conditions (our study)
